# Differential Methylation of the Oxytocin Receptor Gene in Patients with Anorexia Nervosa: A Pilot Study

**DOI:** 10.1371/journal.pone.0088673

**Published:** 2014-02-11

**Authors:** Youl-Ri Kim, Jeong-Hyun Kim, Mi Jeong Kim, Janet Treasure

**Affiliations:** 1 Department of Psychiatry, Inje University, Seoul Paik Hospital, Seoul, Republic of Korea; 2 Indang Institute of Molecular Biology, Inje University, Seoul, Republic of Korea; 3 School of Biological Sciences, Inje University, Gimhae, Republic of Korea; 4 Section of Eating Disorders, Department of Psychological Medicine, King's College London, Institute of Psychiatry, London, United Kingdom; Vanderbilt University Medical Center, United States of America

## Abstract

**Background and Aim:**

Recent studies in patients with anorexia nervosa suggest that oxytocin may be involved in the pathophysiology of anorexia nervosa. We examined whether there was evidence of variation in methylation status of the oxytocin receptor (OXTR) gene in patients with anorexia nervosa that might account for these findings.

**Methods:**

We analyzed the methylation status of the CpG sites in a region from the exon 1 to the MT2 regions of the OXTR gene in buccal cells from 15 patients and 36 healthy women using bisulfite sequencing. We further examined whether methylation status was associated with markers of illness severity or form.

**Results:**

We identified six CpG sites with significant differences in average methylation levels between the patient and control groups. Among the six differentially methylated CpG sites, five showed higher than average methylation levels in patients than those in the control group (64.9–88.8% vs. 6.6–45.0%). The methylation levels of these five CpG sites were negatively associated with body mass index (BMI). BMI, eating disorders psychopathology, and anxiety were identified in a regression analysis as factors affecting the methylation levels of these CpG sites with more variation accounted for by BMI.

**Conclusions:**

Epigenetic misregulation of the OXTR gene may be implicated in anorexia nervosa, which may either be a mechanism linking environmental adversity to risk or may be a secondary consequence of the illness.

## Introduction

Anorexia nervosa (AN) is characterized by abnormal eating patterns, low body weight, and weight and shape concerns. Other features of the illness often include anxiety, social difficulties, and repetitive and rigid behaviors. Genetic factors contribute to the risk of developing AN [1] as do a variety of environmental risk factors [2]. Four critical environmental periods are thought to contribute to alter the neural, physiological, and behavioral aspects of brain function [3]. These include the prenatal period, the neonatal period, early childhood, and adolescence. Adversities in all of these phases of development have been found in patients with AN.

Several studies suggest that factors within the prenatal and perinatal environment may increase the risk of developing an eating disorder. For example, low vitamin D levels during pregnancy are associated with an increased risk of developing an eating disorder [4]. High levels of maternal anxiety during early development have also been implicated [5]. However, a systematic review and meta-analysis concluded that evidence supporting premature birth and obstetric complications as risk factors is weak [6]. Thus, there is some uncertainty about the role of perinatal factors in increasing the risk of an eating disorder. There is also evidence that early childhood factors can increase the risk [2]. Some of these factors produce risk through interaction with genetic factors. For example, a European study found that siblings with the short form of the serotonin transporter were more susceptible to develop AN in the context of early parenting difficulties [7]. Further studies in general populations have also found a similar interaction effect with other markers of environmental adversity (e.g., childhood trauma) [8] and life events [9]. Finally, the onset of AN is during adolescence. It is possible that some of the precipitating factors such as bullying manifest as fat talk [10], [11] or the secondary consequences of the illness (starvation, fasting, and feasting) may impact brain development and later behavior. In a new maintenance model of AN, secondary consequences of starvation are thought to cause the illness to persist by further impairing social processes, emotional regulation, and cognitive flexibility [12], [13].

Anomalies in social and emotional development have been linked to oxytocin systems. There has been particular interest in the oxytocin receptor (OXTR) gene as certain single nucleotide polymorphisms (SNPs) have been linked to empathy, trust, and maternal behavior [14], [15], [16] and interact with exposure to life events to increase the risk of anxiety and depression in females [17]. This is in part due to alterations in brain activation in the ventral striatum [18]. Epigenetic mechanisms such as DNA methylation may underpin the action of some of the interactions between environmental risk factors and oxytocin mechanisms. Hypermethylation of the OXTR gene can suppress its expression [19], [20]. Elevated methylation of the CpG site 934 bp upstream of the translation initiation site is correlated with decreased mRNA level of the OXTR gene in the brain cortex tissue of autism cases [21]. The level of DNA methylation of the OXTR gene is associated with variations in social perception in patients with autistic spectrum disorders [22]. DNA methylation of the OXTR gene and reduced levels of oxytocin are associated with callous unemotional traits and conduct disorder [23]. Thus, methylation status in the MT2 region of the OXTR gene reflects the state of expression of the gene and social functioning.

Oxytocin systems may be of relevance in the psychopathology of eating disorders. A recent review synthesised evidence for abnormal oxytocin functioning in patients with AN [24]. Cerebrospinal fluid levels of oxytocin decrease during the starvation phase of AN [25], [26], [27]. Nocturnal serum oxytocin levels in patients with AN are also reduced [28]. Lawson [29] reported that the release of oxytocin increases in response to a meal in the acute state of AN and decreases after recovery. Anomalies in oxytocin secretion are correlated with severity of the disordered eating psychopathology and with the level of activation in the brain circuitry in response to images of food [29]. It is possible that there are both state and trait anomalies in oxytocin function in patients with AN, and anomalies in OXTR function may account for some of these anomalies.

An additional thread of research in the literature that makes oxytocin of relevance is the possible association between autism and AN. Gillberg [30] suggested that there may be an association between developmental traits such as low empathy and later development of an eating disorder. The two conditions share traits such as difficulties in social cognition, rigidity, and a focus on detail [31]. Several lines of evidence summarized in recent reviews describe similar patterns of neuropsychological functioning and shared traits [32]–[34]. People with eating disorders have higher than normal scores on the autism spectrum quotient (AQ), a questionnaire developed as a self-report measure of typical autism spectrum traits and summarized in a systematic review [35] and recent studies [36]–[38]. It is possible that abnormalities in the oxytocin system may account for these shared traits.

A recent review summarized the evidence suggesting that AN and other psychiatric disorders may be associated with changes in the DNA methylation profile [39]. Research into the epigenetics of eating disorders is at a preliminary stage. One study found global DNA hypomethylation in patients with AN [40] but a more recent small study failed to find differences in the methylation of highly repetitive long interspersed nuclear element sequences [41]. No studies have examined genome-wide epigenetic modifications, as most have investigated candidate gene sites relating to reward and appetite and metabolic function. Anomalies in DNA methylation in the promoter regions of alpha synuclein [40], the dopamine transporter, and the dopamine 2 receptor D2 (DRD2) [42] have been reported in patients with AN, and atrial natriuretic peptide for bulimia nervosa [43]. Normal patterns of methylation have been found for the following candidate genes in AN: proopiomelanocortin [44], leptin, serotonin transporter gene (SERT/SLC6A4), brain derived neurotrophic factor, DRD2 [45], D4 receptor gene [42], vasopressin [46], and insulin-like growth factor [41], in bulimia nervosa for the dopamine D2 DRD2 receptor [47] and glucocorticoid receptor gene (NR3C1) [48]. Nutrition has a major impact on epigenetic processes and it is possible that differences in methylation may result from dietary and metabolic changes [49], [50].

The aim of this study was to examine whether there is evidence of anomalies in methylation status of the OXTR gene in patients with AN. Our first hypothesis is that patients with AN show anomalies in methylation status of the OXTR gene. Our second hypothesis is that methylation status is associated with markers of disease severity such as the severity of weight loss and the level of psychopathology. A third hypothesis is that the pattern of methylation in the MT2 region of the OXTR gene in patients with AN is similar to that seen in autistic disorders [21] and that the methylation status might be linked to the intensity of autistic traits.

## Materials and Methods

### Participants

Characteristics of the sample are shown in Table 1. Fifty-one women (15 patients with AN and 36 healthy university students) were recruited. The patients with AN were recruited from the Eating Disorders Clinic at Seoul Paik Hospital, Seoul, South Korea, and the control group was recruited from an advertisement at a women's university in Seoul, South Korea. The diagnosis of AN was confirmed by the Structured Clinical Interview from the Diagnostic and Statistical Manual of Mental Disorders, Fourth Edition [51]. Exclusion criteria for patients were: active substance use disorder, diagnosis of a psychotic disorder (schizophrenia, schizoaffective, psychosis not otherwise specified), and diagnosis of autism or Asperger's syndrome. The main inclusion criteria for controls were: healthy females without a history of medical or psychiatric illnesses and a minimum of 18 years of age. All subjects were nonsmokers, heterosexual, nulliparous, and were not taking any medications (including the contraceptive pill). The healthy controls were tested during the follicular phase of their menstrual cycle (approximately days 4–12). None of the patients was menstruating. This study protocol was approved by the Institutional Review Board of Seoul Paik Hospital (IIT-2012-096). All participants provided written informed consent prior to participation in the study. Consent was provided by both the patients and their guardians in the case of patients <17 years of age.

**Table 1:** Characteristics of the study population.

	AN (*n* = 15)	Controls (*n* = 36)	*t (df = 49)*	*p*
Age	24.73(10.73)	22.14(2.17)	−1.402	0.167
BMI	15.06 (2.58)	21.04 (2.23)	8.332***	0.000
EDE-Q				
Restraint	2.28(2.12)	0.82 (1.00)	3.284**	0.002
Eating Concern	1.65(1.93)	0.47 (0.85)	3.197**	0.002
Weight Concern	2.20(1.54)	1.30 (1.15)	2.497*	0.016
Shape Concern	2.63(1.77)	1.97 (1.36)	1.500	0.140
Global	2.19(1.67)	1.14 (0.97)	2.936**	0.005
AQ				
Social Skill	4.27 (1.83)	2.68 (1.79)	−2.849**	0.006
Attention Switching	4.33 (2.32)	4.26 (1.38)	−0.129	0.898
Attention to Detail	5.60 (2.06)	3.85 (2.16)	−2.642*	0.011
Communication	3.00 (1.93)	1.71 (1.53)	−2.520*	0.033
Imagination	2.73 (1.79)	2.18 (1.51)	−1.125	0.607
Total	19.93 (5.79)	14.68 (4.28)	−3.547**	0.001
BDI	24.50 (16.31)	7.36 (6.78)	−5.026***	<0.001
STAI_State	57.08 (18.19)	43.48 (11.14)	−3.032**	0.004
STAI_Trat	55.75 (17.76)	43.91 (11.19)	−2.664*	0.011

Data are means (standard deviation).

AN, anorexia nervosa; BMI, body mass index; EDE-Q, Eating Disorder Examination Questionnaire; AQ, Autism-Spectrum Quotient; BDI, Beck Depression Inventory, STAI, Spielberger State and the Trait Anxiety Inventory.

** *p*<0.01, *** *p*<0.001.

### Eating Disorder Examination self-report version Questionnaire (EDE-Q) [52]

The EDE-Q assesses the main behavioral features of an eating disorder over the past 28 days. The questionnaire consists of 36 items on a 7-point forced choice rating scale. It measures weight, shape, eating concerns, and dietary restraint. We used the standardized Korean version of the 12th edition of the EDE-Q, which has high internal consistency and good 2 week reliability

### Autism Spectrum Quotient (AQ) [53]

The AQ is a 50-item, self-administered questionnaire that assesses the degree to which adults with normal intelligence have autism spectrum traits. It has five sub-scales, including social skill, attention switching, attention to detail, communication, and imagination.

### Other measurements

Depression and anxiety were assessed in each subject using the standardized Korean versions of the Beck Depression Inventory (BDI) [54] and the Spielberger State and Trait Anxiety Inventory (STAI) [55], respectively.

### Analysis of methylation status

**Figure 1:**
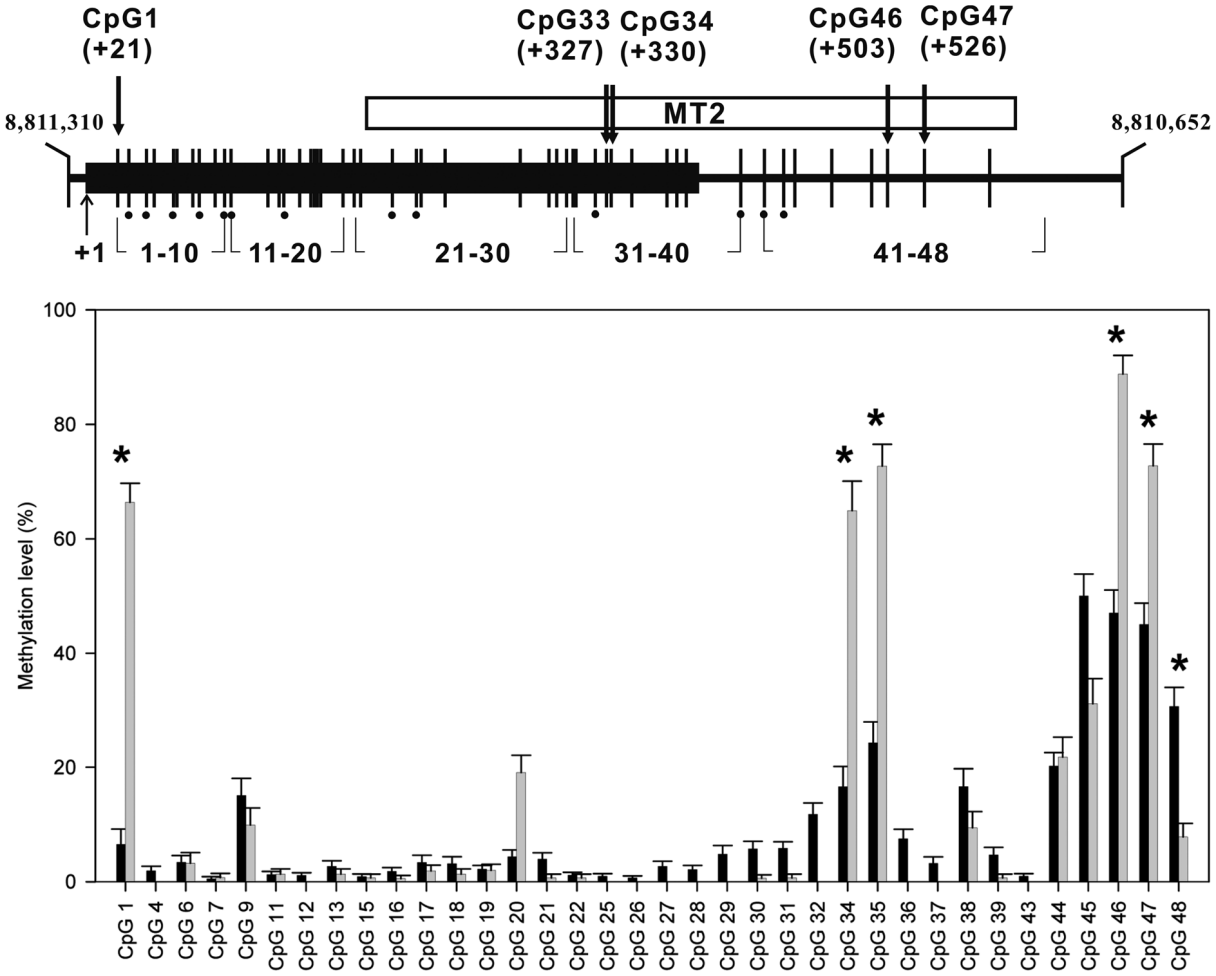
Profile of the average methylation levels at the CpG sites analyzed. The region of the OXTR gene analyzed in this study is represented by a thick horizontal line (top). A total of 48 CpG dinucleotides (vertical lines) were bisulfite sequenced, which covered all CpG sites in exon 1 (black box) and the MT2 region [20] (white box). The CpG sites were counted from transcription start site (TSS) (+1). The CpG sites that overlapped with the single nucleotide polymorphisms registered in the dbSNP database (www.ncbi.nlm.nih.gov/projects/SNP/) were excluded from the statistical analysis (dots below the CpG sites). Those CpG sites with high methylation levels in patients with anorexia nervosa (AN) are indicated by arrows and the relative positions from the TSS of the OXTR gene. The average methylation levels at each CpG site in the control (black) and anorexia nervosas (AN) (grey) groups are shown with the standard error of the mean (bottom). CpG sites that showed significantly different average methylation levels between the AN and control groups are indicated by asterisks.

Genomic DNA was purified from buccal cells collected with DNA Buccal Swabs (Isohelix, Kent, UK) using a PureLink Genomic kit (Invitrogen, Carlsbad, CA, USA). We employed buccal cells for the methylation analysis to avoid the homogeneity issue of peripheral blood mononuclear cells [19], [56] and to take advantage of their common origin with brain tissue. The purified genomic DNA (200 ng) was treated with bisulfite according to the manufacturer's protocol (EpiTect Bisulfite Kit, Qiagen, Valencia, CA, USA). A region of the OXTR gene corresponding to nucleotide positions 8,810,652–8,811,310 of chromosome 3 in the GRCh37.p11 coordinates (Fig. 1) was amplified by the polymerase chain reaction (PCR) using bisulfite-treated DNA as the template. The PCR primers were designed with Methyl Primer Express software v1.0 (Applied Biosystems, Foster City, CA, USA), and their nucleotide sequences were 5′ TAAATTTATTTGTTAAGGTTTTGGG 3 and 5′ TTCCCAAACCCTAACATAAAC 3′. PCR was conducted using the following reaction conditions: one cycle: 95°C for 15 min, 40 cycles: 95°C for 45 s, 55°C for 45 s, followed by 72°C for 1.5 min, and a final extension at 72°C for 10 min. The correct PCR product with a length of 659-bp was collected by 1.5% agarose gel electrophoresis, cloned with pDrive vector (Qiagen), and then transformed into *E. coli* EC100 (Epicentre, Charlotte, NC, USA) by electroporation. The methylation status of each CpG site was determined by nucleotide sequencing of the plasmid DNA purified from each *E. coli* colony with a 3730xl DNA Analyzer (Applied Biosystems). At least 15 clones per subject were sequenced, and their nucleotide sequences were compiled with BISMA software [57] for quality control. Sequences with a bisulfite conversion rate >95% were included, and sequences clonally amplified from the same genomic template were excluded in the final alignments. The BISMA default values were used for all other parameters. The methylation level for the individuals at each of the CpG sites was determined using nucleotide sequences that survived quality control filtering (n≥10) by dividing the number of methylated CpG sites by the total number of CpG sites analyzed at that locus. These values were used to calculate an average methylation level for the AN and control groups. Average methylation levels at each CpG site were compared between the AN and control groups with Student *t*-tests. CpG sites were considered significantly differentially methylated if the p-value was <0.01, and the difference in average methylation levels between the AN and control groups was >20%. CpG sites with a methylation level >65% was considered highly methylated [58]. CpG sites that overlapped with SNPs registered in the SNP database (www.ncbi.nlm.nih.gov/projects/SNP/) were excluded from the final statistical analysis as a confounding factor.

### Statistical analysis

The clinical and demographic data between the AN and healthy control (HC) groups were compared using paired *t*-tests. After Pearson's correlation analysis to assess the relationship between methylation status of the OXTR gene and clinical variables (BMI, eating disorders psychopathology, and other clinical variables), a linear regression analysis was conducted for each CpG site to select factors affecting methylation status of the OXTR gene. P values <0.05 were considered significant, and two-tailed tests were used. A Bonferroni correction was applied if multiple testing is used. Analyses were performed using SPSS 19.0 (SPSS Inc., Chicago, IL, USA).

## Results

### Subject characteristics

The demographic and group characteristics of the participants are shown in Table 1. Mean age and IQ level were similar in the AN and HC groups. As expected, significant differences were observed in BMI (kg/m2) and the EDE-Q scales. The AN group had higher scores on the BDI, the STAI, and on the AQ total subscales of Social Skill, Attention, and Communication than those in the HC group.

### Differential methylation of the OXTR promoter region between the AN and HC groups

We analyzed the methylation status of the CpG sites within the region of the OXTR gene from +1 bp to +582 bp (Fig. 1), which includes the first exon and the MT2 region [20] using bisulfite sequencing. The methylation pattern of the CpG sites was compared in buccal cells from the AN and HC groups. The average methylation levels at the CpG sites for the AN and HC groups are shown in Fig. 1. All CpG sites in exon 1 (CpG 1 to CpG 39) had low levels of methylation (<30% of the average methylation level) in the HC group, with slightly elevated methylated CpG 35 (24.3%), and CpG sites 45–48 in the intron part of the MT2 region were intermediately methylated (30.1–50.0%). We found six CpG sites with significantly different methylation patterns between the AN and control groups (P<0.01) (average methylation level >20%). In particular, CpG 1, CpG 34, CpG 35, CpG 46, and CpG 47 were highly methylated in the AN group (average methylation levels ≥65%). The average methylation levels in the HC group were at least 25% lower (Table 2). We focused on these five CpG highly methylated sites for further statistical analyses.

**Table 2:** Average methylation levels of the CpG sites highly methylated in the anorexia nervosa (AN) group.

	Position	AN (n = 15)	Controls (n = 36)	*t* (df = 49)	*p*
CpG 1	21	66.4 (12.7)	6.6 (16.0)	−12.877***	<.001
CpG 34	327	64.9 (19.8)	16.7 (20.8)	−7.663***	<.001
CpG 35	330	72.7 (14.7)	24.3 (21.9)	−7.834***	<.001
CpG 46	503	88.8(12.5)	47.0 (24.2)	−6.334**	<.001
CpG 47	526	72.7(14.9)	45.0 (22.3)	−4.417**	<.001

Data are means (standard deviation).

Position: relative position from the transcription start site of the oxytocin receptor (OXTR) gene.

** *p*<0.01, *** *p*<0.001.

### Relationship between methylation levels of the OXTR promoter and severity of the disordered eating psychopathology

We examined the association between the five highly methylated CpG sites and clinical features including age, BMI, EDE-Q, AQ, BDI, and STAI (Table 3). Across all participants, the individual methylation levels at all CpG sites correlated negatively with BMI (all p-values <0.05). A Bonferroni correction was applied in the correlation analysis due to multiple testing with the EDE-Q and AQ (alpha  =  0.05/6). The restraint subscale and global scores were positively correlated with methylation level at CpG 1 (r = 0.499, p<0.001 in the restraint subscale; r = 0.382, p = 0.006 in the global subscale). The communication subscale and total score for autism traits were positively correlated with methylation level at CpG 1 (r = 0.414, p = 0.003 in the communication subscale; r = 0.406, p = 0.004 in the total subscale). Depression state on the depression scale was positively correlated with methylation level at CpG 1 (r = 0.425, p = 0.004) and CpG 35 (r = 0.514, p<0.001), and anxiety on the anxiety scale was positively correlated with methylation level at CpG 35 (r = 0.433, p<0.001 for state anxiety; r = 0.428, p = 0.003 for trait anxiety). No correlation was observed between the methylation levels of the CpG sites and clinical variables in the AN group only or in the HC group only.

**Table 3:** Correlations coefficients between methylation levels and severity of disordered eating psychopathology.

		CpG 1	CpG 34	CpG 35	CpG 46	CpG 47
**AGE**	*r*	0.262	0.159	0.195	0.080	0.035
	*p*	0.063	0.266	0.170	0.576	0.809
**BMI**	*r*	−0.700^*^	−0.681^*^	−0.587^*^	−0.415^*^	−0.351
	*p*	<0.001	<0.001	<0.001	0.002	0.011
**EDE**						
Restraint	*r*	0.499^*^	0.211	0.351	0.325	0.256
	*p*	<0.001	0.138	0.011	0.020	0.070
Eating Concern	*r*	0.390^*^	0.240	0.328	0.259	0.252
	*p*	0.005	0.090	0.019	0.066	0.087
Weight Concern	*r*	0.282	0.191	0.297	0.196	0.100
	*p*	0.045	0.179	0.034	0.169	0.486
Shape Concern	*r*	0.190	0.135	0.269	0.122	0.101
	*p*	0.182	0.345	0.056	0.396	0.481
Global	*r*	0.382^*^	0.216	0.348	0.253	0.197
	*p*	0.006	0.128	0.012	0.074	0.167
**AQ**						
Social Skill	*r*	0.307	0.330	0.292	0.190	0.094
	*p*	0.032	0.021	0.042	0.191	0.522
Attention Switching	*r*	0.008	−0.004	0.027	−0.074	0.036
	*p*	0.958	0.981	0.853	0.616	0.806
Attention to Detail	*r*	0.202	−0.020	0.338	0.234	0.254
	*p*	0.164	0.889	0.017	0.106	0.078
Communication	*r*	0.414^*^	0.208	0.293	0.300	0.277
	*p*	0.003	0.151	0.041	0.036	0.054
Imagination	*r*	0.160	0.161	0.173	0.062	0.039
	*p*	0.273	0.270	0.233	0.674	0.791
ASQ_Total	*r*	0.406^*^	0.243	0.431^*^	0.279	0.269
	*p*	0.004	0.093	0.002	0.053	0.062
**BDI**	*r*	0.425^*^	0.352	0.514^*^	0.291	0.337
	*p*	0.004	0.018	<0.001	0.052	0.024
**STAI**						
State	*r*	0.314	0.109	0.433^*^	0.174	0.359
	*p*	0.046	0.476	0.003	0.254	0.015
Trait	*r*	0.290	0.203	0.428^*^	0.130	0.273
	*p*	0.053	0.181	0.003	0.395	0.070

BMI, body mass index; EDE-Q, Eating Disorders Examination-Questionnaire; AQ, Autism-Spectrum Quotient; BDI, Beck Depression Inventory; STAI, Spielberger State and the Trait Anxiety Inventory

A Bonferroni correction was applied (alpha  =  0.05/6). * p < 0.008.

A multiple regression analysis was performed with methylation levels at candidate CpG sites as the dependent variable to identify the contribution of the confounders to methylation level, with age, BMI, EDE-Q global, AQ total, BDI, and STAI as independent variables (Table 4). When controlled for these factors, BMI and eating disorder psychopathology were the main determinants of methylation level at CpG 1 (β = −0.756, p<0.001 in BMI; β = 0.436, p = 0.002 in EDE-Q global). At CpG 34, BMI, eating disorder psychopathology, and anxiety were the main determinants of methylation level (β = −0.845, p<0.001 in BMI; β = 0.368, p = 0.008 in EDE-Q global; β = -0.803, p<0.001 in anxiety). At CpG 35 and 47, BMI was the main determinant of methylation level at CpG 35 (β = −0.575, p = 0.001), and at CpG 47 (β = −0.469, p = 0.015).

**Table 4:** Regression models for oxytocin receptor (OXTR) methylation across participating women.

Model	CpG 1		CpG 34		CpG 35		CpG 46		CpG 47	
R^2^	0.648		0.646		0.522		0.221		0.288	
*P*	<0.001		<0.001		<0.001		0.198		0.063	
Factors	*Beta*	*p*	*Beta*	*p*	*Beta*	*p*	*Beta*	*p*	*Beta*	*p*
Age	0.124	0.259	0.021	0.850	−0.019	0.882	0.064	0.695	−0.151	0.334
BMI	−0.756^***^	<0.001	−0.845^***^	<0.001	−0.575^***^	<0.001	−0.327	0.098	−0.469*	0.015
EDE-Q global	0.436^**^	0.002	0.368**	0.008	0.234	0.134	0.244	0.219	0.188	0.320
AQ total	0.039	0.743	−0.072	0.548	0.156	0.266	0.051	0.774	0.082	0.629
BDI	−0.120	0.563	0.015	0.943	−0.054	0.825	0.219	0.479	−0.184	0.534
STAI_State	−0.376	0.057	−0.803***	<0.001	−0.161	0.476	−0.184	0.523	0.169	0.539
STAI_Trait	0.324	0.132	0.553*	0.013	0.331	0.186	−0.099	0.754	0.102	0.735

BMI, body mass index; AN, anorexia nervosa; EDE-Q, Eating Disorder Examination Questionnaire; AQ, Autism-Spectrum Quotient; BDI, Beck Depression Inventory; STAI, Spielberger State and the Trait Anxiety Inventory.

* *p*<0.05, ** *p*<0.01, *** *p*<0.001.

## Discussion

We examined whether there was evidence that variation in methylation status of the OXTR gene is associated with patients with AN. Our first hypothesis was confirmed in that individuals with AN had different levels of methylation compared to those in the control sample. Thus, methylation status at several sites within the OXTR gene, in particular, at CpG 1, 34, 35, 46, and 47 showed high levels of methylation in patients with AN but low or intermediate levels of methylation in healthy women. Our second hypothesis was also confirmed in that methylation status was associated with markers of disease severity such as weight loss severity. Our third hypothesis was unsupported in that autistic traits were not included in the final regression model of OXTR gene methylation.

In addition to the 4 CpG sites located in the MT2 region (CpG 33, 34, 45, and 46), we also identified a CpG site at + 21 from the TSS (CpG 1), which was highly methylated in patients with AN. The extent of methylation correlated with markers of severity (BMI) and eating disorder psychopathology. Methylation at this CpG site has not been implicated in epigenetic regulation of the OXTR gene, so further research may be warranted. The presence of highly methylated CpG sites in the OXTR promoter region in patients with AN may act to suppress OXTR gene expression [20], [21], [59]. This might explain some of the anomalies in oxytocin function found by Lawson and colleagues [27], [29]. High levels of methylation of the OXTR gene are associated with callous unemotional traits in young males presenting with conduct problems [23]. It is possible that this methylation may account for anomalies in social processing seen in AN [60], [61].

The association between methylation and markers of disease severity such as weight (BMI) suggest that this could be causally related to AN. However, it is also possible that illness-related factors such as weight loss and changes in dietary composition may have contributed to the increased methylation. Nutrients and bioactive food components are known to impact epigenetic phenomena such as DNA methylation and histone modifications [62]. Folic acid, a cofactor in one-carbon metabolism, regulates epigenetic processes, and folate deficiency provokes reduced DNA methylation [63].

Our study had some limitations. First, we had a low number of subjects in the AN group and all were included in the acute, malnourished state. Thus, replication is required in larger, independent samples and after weight and full recovery. The second limitation is that we measured methylation status of the OXTR gene in buccal cells. It is uncertain whether the methylation pattern in buccal cells reflects the pattern in brain tissue even though they have a common ectodermal origin. It would be of interest to examine whether these findings might be replicated in other tissues (brain tissue if possible would be optimal). The third limitation is that we did not assess smoking behavior or folate levels, which impact on methylation, and that may differ between controls and patients with eating disorders.

It is possible that environmental factors during development may account for the high levels of methylation within the OXTR gene. An alternative hypothesis is that the profound physiological perturbations of the illness may themselves contribute to anomalous methylation. Further research will be needed to explore this potential risk factor.

In summary, our data suggest that epigenetic mechanisms in the OXTR gene may be implicated in AN. We identified four highly-methylated CpG sites (CpG 34, CpG 35, CpG 46, and CpG 47) in the MT2 region that are known to regulate expression of the OXTR gene in patients with AN. Methylation level was inversely related to BMI.
